# Abundance, zoonotic potential and risk factors of intestinal parasitism amongst dog and cat populations: The scenario of Crete, Greece

**DOI:** 10.1186/s13071-017-1989-8

**Published:** 2017-01-25

**Authors:** Despoina Kostopoulou, Edwin Claerebout, Dimitrios Arvanitis, Panagiota Ligda, Nikolaos Voutzourakis, Stijn Casaert, Smaragda Sotiraki

**Affiliations:** 1 Laboratory of Parasitology, Faculty of Veterinary Medicine, Ghent University, Salisburylaan 133, Merelbeke, B-9820 Belgium; 2 Veterinary Research Institute - Hellenic Agricultural Organization Demeter, Thermi, Thessaloniki 57001 Greece

**Keywords:** Intestinal parasites, Companion animals, Zoonotic, Molecular analyses

## Abstract

**Background:**

The objectives of this study were to evaluate the prevalence and infection intensity of intestinal parasites in different dog and cat populations in Crete, Greece, estimate the zoonotic risk and identify risk factors.

**Methods:**

Faecal samples from shelter, household and shepherd dogs and shelter and household cats were analyzed using sedimentation/flotation techniques. *Giardia* and *Cryptosporidium* were detected by a quantitative direct immunofluorescence assay (IFA). PCR and sequencing was performed to evaluate the zoonotic potential of *Giardia* and *Cryptosporidium* positive samples.

**Results:**

Totals of 879 dog and 264 cat faecal samples were examined. In dogs, the overall prevalence was 25.2% (CI: 22.4–28.1) for *Giardia* spp.; 9.2% (CI: 7.3–11.1) for *Ancylostoma/Uncinaria* spp.; 7.6% (CI: 5.9–9.4) for *Toxocara* spp.; 5.9% (CI: 4.4–7.5) for *Cryptosporidium* spp.; 4.6% (CI: 3.2–5.9) for *Cystoisospora* spp.; 2.7% (CI: 1.7–3.8) for *Toxascaris leonina*; 1.7% (CI: 0.9–2.6) for *Capillaria* spp.; 0.8% (CI: 0.2–1.4) for taeniid eggs; 0.2% (CI: 0–0.5) for *Dipylidium caninum*; and 0.1% (CI: 0–0.3) for *Strongyloides stercoralis.* In cats, the prevalence was 20.5% (CI: 15.6–25.3) for *Giardia* spp.; 9.5% (CI: 5.9–13.0) for *Cystoisospora* spp.; 8.3% (CI: 5.0–11.7) for *Toxocara* spp.; 7.6% (CI: 4.4–10.8) for *Ancylostoma/Uncinaria* spp.; 6.8% (CI: 3.8–9.9) for *Cryptosporidium* spp.; 4.2% (CI: 1.8–6.6) for *Capillaria* spp.; 0.8% (CI: 0–1.8) for taeniid eggs; and 0.4% (CI: 0–1.1) for *Hammondia/Toxoplasma*. Concerning the risk factors evaluated, there was a negative association between age and *Giardia* infection and between age and *T. leonina* infection intensity for dogs. Sequencing results revealed the presence of mainly animal-specific *G. duodenalis* assemblages C and D in dogs and assemblages F, C and BIV-like in cats, with only a limited number of (co-)infections with assemblage A. As for *Cryptosporidium*, the dog-specific *C. canis* and the pig-specific *C. scrofarum* were detected in dogs and the cat-specific *C. felis* was detected in cats.

**Conclusions:**

High levels of parasitism in both dogs and cats were recorded. *Giardia* was the most prevalent parasite in all dog and cat populations except for shepherd dogs. Genotyping results suggest a limited zoonotic risk of *Giardia* and *Cryptosporidium* infections from dogs and cats in Crete. Taeniid eggs were more prevalent in shepherd dogs suggesting access to carcasses and posing a threat for cystic echinococcosis transmission. Infection rates of *Toxocara* spp. in both dogs and cats show that companion animals could be a significant source of infection to humans.

**Electronic supplementary material:**

The online version of this article (doi:10.1186/s13071-017-1989-8) contains supplementary material, which is available to authorized users.

## Background

Intestinal parasite infections are still abundant in companion animals, despite all the highly efficient drug formulations available and the control measures taken by owners and veterinarians [1–8]. Moreover, parasites are responsible for some of the most important and well-recognized zoonoses transmitted from companion animals to man globally such as *Giardia* spp., *Cryptosporidium* spp., *Toxocara* spp., hookworms and *Echinococcus granulosus* [9–13].

Nowadays, changes due to climate alterations and social behaviour that affect humans’ lives and consequently the lives of the animals which live close to them [14, 15], alter the interactions between humans and pathogens leading to (re)emergence of several diseases, including zoonotic ones [16, 17].

The distribution of zoonoses associated with companion animals is highly affected by animals’ movements (between regions, countries and continents) which in fact are the means to relocate pathogens and vectors they harbour. The above is becoming more and more important since human travel continues to increase in parallel with the population and financial status increase, and when humans travel, they often take their companion animals, particularly dogs.

All the above is in fact unfolding the reasons why it is crucial to fill the gaps on the current distribution of these diseases in a constantly changing environment and to describe the risks associated with pet infection in order to assure their well-being and to prevent the free movement of zoonotic pathogens.

The aim of our study was to investigate the presence and infection intensity of intestinal parasites in dogs and cats, the risk factors (such as lifestyle, veterinary care, etc.) that influence those infections and their zoonotic potential. This was done by performing a cross-sectional epidemiological study within a defined animal/human community, i.e. the island of Crete, as a case scenario.

## Methods

### Populations studied

**Fig. 1:**
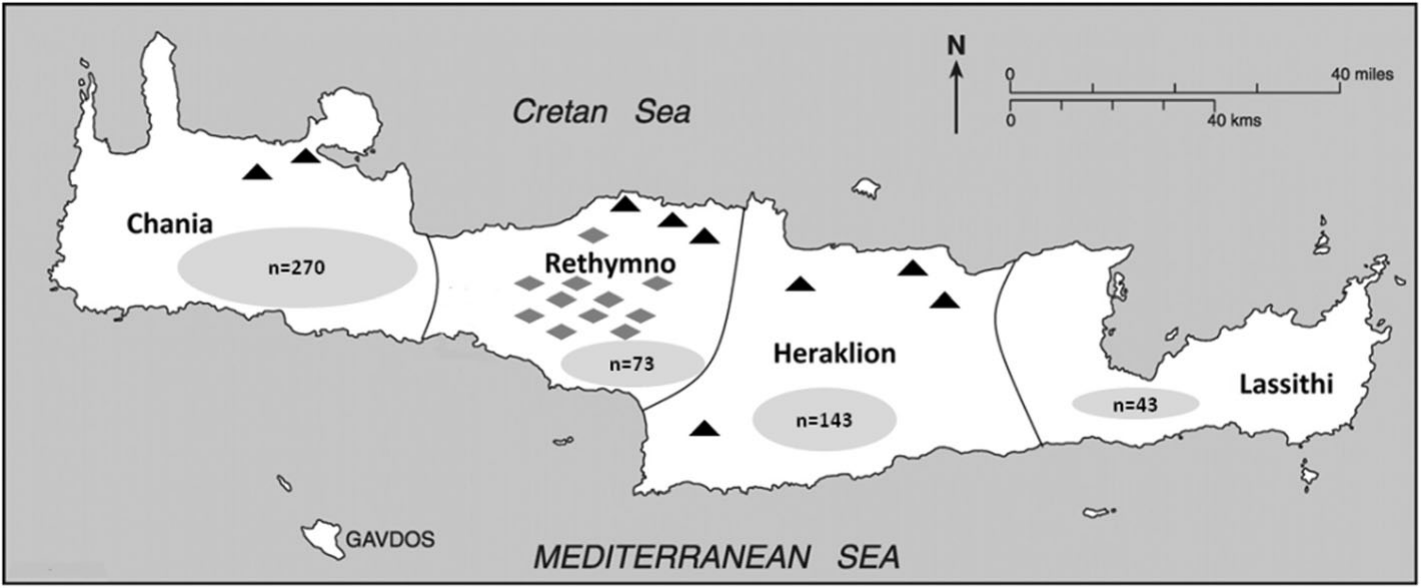
Map of Crete demonstrating the locations of different sample points per animal population category. *Key*: triangles: shelter dogs/cats; rhombi: shepherd dogs; gray ellipses: household dogs/cats with the number of animals sampled

Faecal samples were collected from different dog populations (shelter, household and shepherd) as well as shelter and household cats in Crete Island in Southern Greece (Fig. 1), from October 2011 to January 2015.

Crete is the largest and most densely populated island of Greece (623,000 residents recorded in 2011) with a population well distributed in urban and rural areas. The island is also a highly popular tourist destination (approximately 3.5 million international tourist passengers’ arrivals in 2013) (Region of Crete: www.crete.gov.gr). Moreover in Crete, in addition to the high number of companion animals, there is a significant livestock and wildlife population (Hellenic Ministry of Rural Development and Food: http://minagric.gr).

Since data on the precise population of pets in the location were not available, the sample size was determined estimating the dog and cat population size as “infinite”. The prevalence of intestinal parasitism in different dog and cat studies in Europe varies enormously depending on the sampled animal population and the diagnostic techniques that were used [3, 6, 18–24]. In this study, in order to calculate the sample size (with a precision of 5% and a 95% confidence interval) we selected to relate our “expected prevalence” values to recent reports of *Giardia* prevalence in Europe. Therefore, the targeted sample size was defined as follows: for household dogs up to 200 dogs (reported prevalence 10–20%); for shelter dogs up to 400 dogs (reported prevalence 20–50%); for household cats 138 cats (reported prevalence < 10%) and for shelter cats 385 cats (reported prevalence 10–50%) [3, 5, 18, 19, 25–28]. For shepherd dogs there is little information available and given the difficulties in approaching and handling such dogs we aimed at collecting the maximum feasible number of samples. In order to achieve the most accurate coverage of the whole island, the animals enrolled in our study were allocated proportionally to the four different counties of the island according to the inhabitant’s population density (Fig. 1).

Individual rectal faecal samples were randomly collected from dogs and cats of all ages with or without intestinal symptoms from 561 households, 11 shelters and 29 sheep and goat farms. After collection, the samples were immediately transported under vacuum [29] to the laboratory where they were stored at 4 °C and examined within 2 days. When a sample was found to be positive by coproscopic analysis for *Giardia* spp. or *Cryptosporidium* spp., it was stored at -20 °C until DNA extraction was performed and molecular genotyping followed.

For every animal/sample, a data-form was completed by interviewing the owner or in case of shelters the person who was responsible for the animals, providing information on age, sex, breed, living conditions (indoors or outdoors), presence of other animals, the presence or absence of diarrhoea (up to maximum 1 month before sampling), if the animal had travelled recently and the antiparasitic treatment plan followed (including time of last treatment). Faecal consistency was recorded for all faecal samples. The consistency of individual faecal samples was scored using the following scale: 1, formed; 2, soft; 3, diarrhoea, 4, haemorrhagic diarrhoea.

### Parasitological techniques

The presence of worm eggs and protozoan oocysts was determined by applying two different methods, i.e. a sedimentation (acid/ether) and a sedimentation/flotation technique (using a saturated sugar salt solution as a flotation fluid with 1.28 specific gravity) [30]. For the detection of *Giardia* spp. and *Cryptosporidium* spp. (oo)cysts a quantitative direct immunofluorescence assay (IFA) based on the commercial MERIFLUOR *Cryptosporidium*/*Giardia* kit (Meridian Diagnostics Inc., Cincinnati, Ohio) was used [31, 32].

### Molecular analyses

DNA was extracted from the positive *Giardia* spp. and *Cryptosporidium* spp. faecal samples using the QIAamp® Stool Mini Kit (Qiagen, Hilden, Germany) according to the manufacturer’s instructions. For the amplification of the *Cryptosporidium* 18S ribosomal RNA gene (rDNA18S) and HSP70 gene, previously described PCR protocols were used [33, 34]. For the identification of *Giardia* DNA, the *Giardia* rRNA 18S gene (rDNA 18S) [35], the β-giardin gene [36], the triose phosphate isomerase (TPI) gene [37] and the glutamate dehydrogenase (GDH) gene [38] were used. Amplification products were visualised on 1.5% agarose gels with ethidium bromide. A positive (genomic DNA from a positive faecal sample) and negative (PCR water) control sample were included in each PCR reaction.

PCR products were purified and sequenced from both strands. PCR products were purified using the Qiaquick PCR purification kit (Qiagen) and fully sequenced using the Big Dye Terminator V3.1 Cycle sequencing Kit (Applied Biosystems, California, USA). Sequencing was performed by an external company (GATC Biotech) using the Big dye Terminator V3.1 Cycle sequencing Kit (Applies Biosystems) and the reactions were analyzed using a 3730xl DNA Analyzer (ThermoFisher Scientific). Sequences were assembled using Seqman 5.0 Software (Lasergene DNASTAR) and were aligned using the Basic Local Alignment Search Tool (BLAST) as well as compared with reference sequences using MegAlign (Lasergene DNASTAR) (Additional file 1). For multilocus genotyping Clustal X, 2.0.11 software was used and reference sequences were selected according to Caccio et al. [39].

### Statistical analysis

Descriptive statistical analyses and multivariate methodologies were performed using the statistical language R [40] and the *pscl* package [41]. Two approaches were applied as follows.

#### Multivariate binary logistic models

The effect of the independent variables (age in months, gender, food, travel, neutering, living conditions, living with other animals, antiparasitic treatment, time between treatment and sampling date, diarrhoea during the last month, faecal score and type) on a sample being or not infected by a parasite was studied through the utilization of multivariate logistic models with forward LR selection. Initially, a test of the full model against a constant only model was performed in order to assess whether there was a statistically significant effect of the examined independent predictors on the response variable through the utilization of the Omnibus Tests of Model Coefficients, which uses the Chi-square test to see if there is a significant difference between the log-likelihood (-2LL) of the baseline model (constant model) and the model with the predictors. In addition, the Hosmer & Lemeshow (H-L) test was performed to test whether the model provides a good fit to the data (Additional file 2: Table S1).

#### Multivariate zero-inflated models

The effect of the independent variables on the parasitic infection intensity (egg/(oo)cyst counts per gram) was studied through the utilization of a zero-inflated negative binomial model [42] due to the excess of zero counts and overdispersion of the data. In this analysis the group of shepherd dogs were not included due to the limited number of samples examined (Additional file 2: Table S2).

## Results

### Dogs

A total of 879 faecal samples from dogs were investigated for the presence of intestinal parasites. Of these samples, 278 were derived from shelter dogs, 529 from household dogs and 72 from shepherd dogs (Table 1). In total, 38.3% of dogs were found harbouring at least one intestinal parasite. Precisely 25.5% were harbouring one parasite, 8.9% two and the rest 3–6 different species. The overall infection rate was 25.2% (CI: 22.4–28.1) for *Giardia* spp.; 9.2% (CI: 7.3–11.1) for *Ancylostoma/Uncinaria* spp.; 7.6% (CI: 5.9–9.4) for *Toxocara* spp.; 5.9% (CI: 4.4–7.5) for *Cryptosporidium* spp.; 4.6% (CI: 3.2–5.9) for *Cystoisospora* spp.; 2.7% (1.7–3.8) for *Toxascaris leonina*; 1.7% (CI: 0.9–2.6) for *Capillaria* spp.; 0.8% (CI: 0.2–1.4) for taeniid eggs*,* 0.2% (CI: 0–0.5) for *Dipylidium caninum*; and 0.1% (CI: 0–0.3) for *Strongyloides stercoralis.* The results for the different dog populations are shown in Table 1.

**Table 1:** Prevalence of intestinal parasites and factors associated with this prevalence in different dog populations. Percentages given for specific parasites refer to percentage of dogs that were found positive for an infection within a category of risk factor

Dog population	Parasite species	Prevalence (%) (95% CI)	Infection intensity	History of diarrhoea	
			Median^a^ (Range)	With diarrhoea	Without diarrhoea
Shelter	All	62.9 (57.3–68.6)		46.0	54.0
	*Giardia*	54.3 (48.5–60.2)	4,450 (100–222,800)	51.6	56.7
	*Cryptosporidium*	14.7 (10.6–18.9)	200 (100–1,400)	7.8	20.7
	*Toxocara* spp.	12.2 (8.4–16.1)	79 (1–12,000)	3.9	19.3
	*T. leonina*	6.1 (3.3–8.9)	153 (2–3,330)	0.8	10.7
	Hookworms	9.7 (6.3–13.2)	31 (3–588)	3.1	15.3
	Capillariidae	0.7 (0.0–1.7)	1.5 (1 & 2)	0.8	0.7
	*Cystoisospora*	7.6 (4.4–10.7)	26 (1–1,800)	7.8	7.3
Household	All	23.8 (20.2–27.4)		25.5	74.5
	*Giardia*	12.9 (10.0–15.7)	10,400 (100–75,800)	20.5	10.4
	*Cryptosporidium*	1.9 (0.7–3.1)	300 (100–40,300)	4.5	1.3
	*Toxocara* spp.	5.1 (3.2–7.0)	29 (2–4,284)	6.8	4.6
	*T. leonina*	0.9 (0.1–1.8)	51 (15–223)	0	1.0
	Hookworms	5.3 (3.4–7.2)	9 (1–423)	1.5	6.6
	Capillariidae	1.9 (0.7–3.1)	4.5 (1–1,246)	2.3	1.8
	*Cystoisospora*	2.5 (1.1–3.8)	7 (1–8,400)	3.8	2.0
Shepherd	All	51.4 (39.8–62.9)		0	100
	*Giardia*	4.2 (0.0–8.8)	11,800 (3,000–122,700)	0	4.2
	*Cryptosporidium*	1.4 (0.0–4.1)	1,200 (1,200)	0	1.4
	*Toxocara* spp.	8.3 (1.9–14.7)	118.5 (27–2543)	0	8.3
	*T. leonina*	2.8 (0.0–6.6)	282.5 (109 & 456)	0	2.8
	Hookworms	33.3 (22.4–44.2)	19 (1–894)	0	33.3
	Capillariidae	4.2 (0.0–8.8)	20 (1–36)	0	4.2
	*Cystoisospora*	8.3 (1.9–14.7)	3.5 (1–41)	0	8.3
	Taeniid	6.9 (1.1–12.8)	16 (3–72)	0	6.9

^a^Median number of cysts/oocysts/eggs per gram of faeces

Among the different canine populations studied, shelter dogs had the highest infection rates. In particular, 62.9% of the shelter dogs were infected with at least one species of endoparasite compared to 51.4% of the shepherd dogs and 23.8% of the household dogs. According to the multivariate binary logistic model analysis, the odds ratio (OR) of *Giardia* infection was higher in shelter dogs than household dogs (11.24 times higher) and shepherd dogs (15.63 times higher). However, based on the multivariate zero-inflated model, among *Giardia*-infected individuals, household dogs had generally higher cyst counts than shelter dogs (OR = 1.602). Regarding *Cryptosporidium*, and according to the multivariate zero-inflated model, the odds ratio in favour of zero *Cryptosporidium* OPG for household dogs was 8.248 times higher than that for shelter dogs, suggesting that household dogs were less prone to *Cryptosporidium* infection than shelter dogs. However, *Cryptosporidium*-positive household dogs shed more oocysts than infected shelter dogs (OR = 12.182). No statistically significant correlations between infection with the other parasites and their living conditions were detected in both models (Table 2).

**Table 2:** Prevalence of intestinal parasites and factors associated with this prevalence in different cat populations. Percentages given for specific parasites refer to percentage of cats that were found positive for an infection within a category of risk factor

Cat population	Parasite species	Prevalence (%)(95% CI)	Infection intensity	History of diarrhoea	
			Median^a^ (Range)	With diarrhoea	Without diarrhoea
Shelter	All	55.9 (43.3–68.6)		15.3	84.7
	*Giardia*	39.0 (26.5–51.4)	5,700 (100–700)	33.3	40.0
	*Cryptosporidium*	11.9 (3.6–20.1)	100 (100–700)	22.2	10.0
	*Toxocara* spp.	10.2 (2.5–7.9)	3 (1–63)	22.2	8.0
	*T. leonina*	0	0	0	0
	Hookworms	5.1 (−0.5–10.7)	7 (5–22)	0.0	6.0
	Capillariidae	5.1 (0.0–10.7)	38 (17–84)	0.0	6.0
	*Cystoisospora*	8.5 (1.4–15.6)	42 (2–2,330)	0.0	10.0
Household	All	33.2 (26.7–39.6)		35.9	64.1
	*Giardia*	15.6 (10.6–20.6)	5,800 (100–248,100)	16.4	13.1
	*Cryptosporidium*	5.4 (2.3–8.5)	400 (100–1,800)	5.5	4.6
	*Toxocara* spp.	7.8 (4.1–11.5)	278.5 (1–2,500)	2.7	10.0
	*T. leonina*	0	0	0	0
	Hookworms	8.3 (4.5–12.1)	72 (1–523)	5.5	10.0
	Capillariidae	3.9 (1.3–6.6)	8.5 (1–1,161)	4.1	3.8
	*Cystoisospora*	9.8 (5.7–13.8)	84.5 (1–6,114)	8.2	9.2

^a^Median number of cysts/oocysts/eggs per gram of faeces

**Fig. 2:**
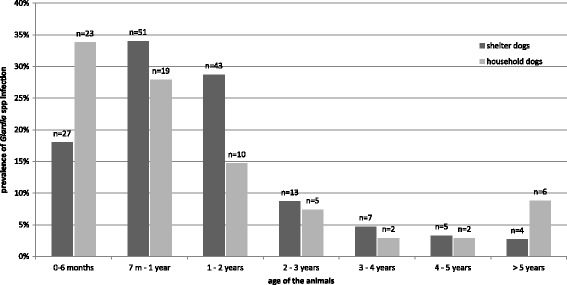
Prevalence of *Giardia* spp. in different dog populations and different age groups

The mean age of the sampled dogs was approximately 3 years (39.5 months ± 41.8, SD). The majority of the dogs were adults (≥ 12 months, *n* = 642), while 229 of them were younger than 12 months and 8 were of unspecified age. There was a significant correlation between age and *Giardia* infection (Fig. 2) and between age and *T. leonina* infection intensity. According to the multivariate binary logistic model analysis, as age increased by one month, the odds of detecting *Giardia* cysts decreased by 1.9% = [(0.981–1) × 100] which is also confirmed by the multivariate zero-inflated model, according which the odds of absence of *Giardia* cysts are increased by one unit increase of age. Similarly, according to the multivariate zero-inflated model, as age increased by one month, the odds of detecting *T. leonina* eggs decreases by 7% = [(0.93–1) × 100]. Regarding the other parasites studied, their correlation with age was not statistically significant.

Of the dogs which had a history of recent diarrhoea, 43.1% were positive for at least one intestinal parasite. However, faecal consistency was not significantly associated with parasitic infection. The statistical analyses showed that signs of diarrhoea (based on faeces consistency) were significantly more often present in younger animals (*U =* 100,667, *P* = < 0.001). Moreover, there was a statistically significant association between the factors “recent record of diarrhoea” and “live with other animals”, (*χ* ^2^_(6, *N* = 1138)_ = 29.495, *P = < *0.001).

On average, all of the dogs sampled received 2.1 anthelmintic treatments/year (range 0–6). The arithmetic mean of anthelmintic treatments/year was 2.3 for household dogs, 2.2 for shelter dogs and 0.5 for shepherd dogs. Information about anthelmintic treatments was not defined in 48 cases (5.5%). The frequency of antiparasitic treatment was also associated with diarrhoea and more specifically, the effect of the odds of one treatment per year increase resulted in a decrease by 0.828 times in the trace of “recent record of diarrhoea”, implying diarrhoea to be caused by parasite infestation. However, the number of antiparasitic treatments/year received was not statistically associated with parasitic infection.

The risk analyses of all the other factors which were evaluated in this study, such as the gender of the animals, their living conditions (indoors/outdoors), the type of food, and recent travelling, showed no statistically significant correlation with parasitic infection. Since almost all shelter dogs had access to the external environment and the shepherd dogs were also living outside, the risk factor “living indoors/outdoors” was assessed only for household dogs. The risk factor “recent travelling” was also not analysed since only 4.2% of the dogs had been travelling during the last months before sampling, including within counties. The same applied for the “type of food” factor, since the majority of the dogs were eating industrial/cooked food and only 28 were fed with raw meat/offal, 64% of these being shepherd dogs.

*Giardia* spp. was the most prevalent parasite in all dogs (25.2%) and also in shelter (54.3%) and household (12.9%) dogs in particular. The range of the cysts being shed by the infected animals varied from 100 to 275,800 cysts per gram of faeces with 6,855 cysts shed on average. In the samples derived from shelter and household dogs, the dog-specific assemblages C and D were dominating, either alone (*n* = 72) or in mixed infections (*n* = 15). A limited number of dogs were infected with assemblage A (*n* = 2), assemblage AI (*n* = 1), assemblage AII (*n* = 1) or a mixture of A with C or D (*n* = 5) or BIV-like and C (*n* = 1) (Table 3). Regarding shepherd dogs, no positive PCR products were sequenced successfully. Multilocus genotyping was performed from one dog sample which was classified as sub-assemblage AI using 3 genetic loci (bg, TPIGEN and GDH). Alignment analysis of the isolate showed 100% homology when compared to reference sequences A5 for bg; A1 for TPIGEN and A1 for GDH [39], resulting in multilocus genotype MLGA1 [43].

**Table 3:** Genotyping results of samples from dogs infected by *Giardia duodenalis* (at all different loci)

No.	Host species	Assemblage type			
		18S	bg	TPI	gdh
1	Shelter Dog	C			
2	Shelter Dog				C
3	Shelter Dog	C	D	C + D	
4	Shelter Dog		D		
5	Shelter Dog	C		BIV-like	
6	Shelter Dog	C			
7	Shelter Dog	C	C	AI	
8	Shelter Dog	C		C	
9	Shelter Dog	C			
10	Household Dog	A			
11	Household Dog	C		C + D	
12	Shelter Dog	D	D		
13	Shelter Dog	C		AI	
14	Shelter Dog	C			C
15	Shelter Dog			D	D
16	Shelter Dog		C	AI + C + D	C
17	Shelter Dog	C		C + D	
18	Household Dog	C		C	C
19	Shelter Dog		D		D
20	Shelter Dog		D		
21	Shelter Dog	D	D	D	D
22	Shelter Dog			C + D	
23	Household Dog	C			
24	Household Dog				
25	Household Dog	D			D
26	Household Dog	D		D	
27	Household Dog	C			
28	Household Dog	C	C	C	C
29	Household Dog	A	AI	AI + C + D	AI
30	Household Dog	C			
31	Household Dog	D	D	D	D
32	Household Dog	D	D	D	D
33	Shelter Dog	C	C	C + D	C
34	Shelter Dog	C	C	C	C
35	Shelter Dog	D	D	D	D
36	Shelter Dog	C	C	C + D	C
37	Shelter Dog	C			
38	Shelter Dog	C	C	C	C
39	Shelter Dog	C		C + D	C
40	Shelter Dog	C			C
41	Shelter Dog				D
42	Household Dog				D
43	Household Dog	C			C
44	Household Dog	D			D
45	Household Dog	C			C
46	Shelter Dog	C		C + D	C
47	Household Dog	C			
48	Shelter Dog	C	D		D
49	Shelter Dog	C			
50	Shelter Dog	C			
51	Shelter Dog	C			D
52	Shelter Dog	D		D	
53	Shelter Dog		D	C + D	D
54	Shelter Dog	C			C
55	Shelter Dog	C			C
56	Shelter Dog	C	C	C + D	C
57	Shelter Dog	C	D	D	C
58	Shelter Dog				
59	Shelter Dog	D			D
60	Shelter Dog	C	C		C
61	Shelter Dog	C			
62	Shelter Dog	C			
63	Shelter Dog	C	C		
64	Shelter Dog	C			
65	Shelter Dog	C			
66	Shelter Dog	C		C	
67	Shelter Dog	C	C		C
68	Shelter Dog	C			
69	Shelter Dog	C			
70	Shelter Dog	C	C		C
71	Shelter Dog	C			D
72	Household Dog				D
73	Shelter Dog	D		D	
74	Shelter Dog	C			
75	Shelter Dog	D			D
76	Shelter Dog				
77	Shelter Dog	D			
78	Shelter Dog				
79	Shelter Dog	C			
80	Shelter Dog	C			
81	Shelter Dog	C			
82	Shelter Dog	C	C		
83	Shelter Dog	D			
84	Household Dog	C			
85	Shelter Dog		AI		
86	Shelter Dog		C		
87	Household Dog		C	C	C
88	Household Dog				
89	Household Dog				
90	Household Dog		D	C + D	
91	Household Dog			D	
92	Shelter Dog			AII	
93	Shelter Dog	A			
94	Shelter Dog	D			
95	Shelter Dog	C			
96	Shelter Dog				D
97	Household Dog				C
98	Shelter Dog				D
99	Shelter Dog				
100	Shelter Dog			D	
101	Shelter Dog				
102	Shelter Dog				
103	Household Dog			D	
104	Household Dog			D	D
105	Household Dog			D	AII
106	Household Dog				C

The PCR results for *Cryptosporidium* positive samples showed that the HSP70 gene amplified 23.6% of the samples, whereas the 18S rDNA gene amplified 5.6%. Sequencing revealed the presence of *Cryptosporidium canis* in 2 household dogs and *C. scrofarum* in a shelter dog.

### Cats

In total, 264 faecal samples from cats were collected; 59 samples from shelters and 205 from owned cats. Unfortunately, it was not possible to reach the target of 385 shelter cats. Overall, 38.1% of the cats were harbouring at least one intestinal parasite. Precisely 26.4% were harbouring one parasite, 8.3% two and the rest 3–4 different species. The prevalence was 20.5% (CI: 15.6–25.3) for *Giardia* spp.; 9.5% (CI: 5.9–13.0) for *Cystoisospora* spp.; 8.3% (CI: 5.0–11.7) for *Toxocara* spp.; 7.6% (CI: 4.4–10.8) for *Ancylostoma/Uncinaria* spp.; 6.8% (CI: 3.8–9.9) for *Cryptosporidium* spp.; 4.2% (CI: 1.8–6.6) for *Capillaria* spp.; 0.8% (CI: 0.0–1.8) for taeniid eggs; and 0.4% (CI: 0–1.1) for *Hammondia/Toxoplasma*. The results among different feline populations are shown in Table 4.

**Table 4:** Genotyping results of samples from cats infected by *Giardia duodenalis* (at all different loci)

No.	Host species	Assemblage type			
		18S	bg	TPI	gdh
1	Shelter Cat	A			
2	Shelter Cat	A			
3	Shelter Cat	A	F	F	
4	Shelter Cat	A			
5	Shelter Cat	A			
6	Household Cat		F	F	F
7	Shelter Cat			BIV-like	
8	Household Cat	A	F		F
9	Household Cat				
10	Household Cat	A	F	F	F
11	Household Cat	A	F	F	F
12	Household Cat	A			
13	Household Cat	A			
14	Household Cat			F	
15	Household Cat				no result
16	Household Cat				C

The mean age of the sampled cats was 3.4 years (40.8 months ± 48.9, SD). The majority of the cats were adults (≥ 12 months, *n* = 161), while 97 of them were younger than 12 months and 6 were of unspecified age.

Among the different feline populations studied, shelter cats had the highest infection rates. Specifically, 55.9% of the shelter cats were infected with at least one species of intestinal parasite compared to 33.2% of the household cats. However, infection rates of the different parasites were not statistically different between different cat populations.

Of the cats which had a history of diarrhoea (30.9%), 32.9% were infected with at least one parasite. On average, all cats sampled received 2.3 anthelmintic treatments/year (range 0–6). The mean number of anthelmintic treatments/year was 1.9 for household cats and 2.7 for shelter cats. Information about anthelmintic treatments was unknown in one case. Only 1.5% of the cats had been travelling during the last months including within counties. No significant associations were found between parasite infections and risk factors or between parasite infections and diarrhoea.

*Giardia* spp. was the most prevalent parasite (20.5%), both in shelter cats (39.0%) and household cats (15.6%). When targeting the 18S rRNA gene, assemblage A was identified in 10 cat samples. In 6 of these samples, no amplification was obtained with the other genes, while in 4 samples only assemblage F was detected in at least one of the other loci. Assemblage F was also found alone in 2 samples. Also, in two different cases, the typing revealed the presence of assemblage BIV-like (*n* = 1) or the dog specific assemblage C (*n* = 1) (Table 4).

Genotyping of *Cryptosporidium* positive samples showed the presence of the feline specific species *Cryptosporidium felis* (*n* = 4).

## Discussion

The infection rates of intestinal parasites detected in this study, revealed a high prevalence of parasitic infections (38.2%) and the presence of different species of endoparasites in both dogs and cats. These infection rates were equally distributed within animal species (38.3% for dogs and 38.1% for cats) involved in the study. With the exception of shepherd dogs, *Giardia* spp. was the most prevalent parasite detected in the dog and cat populations followed by significant prevalences of ascarids, hookworms and taeniid infections. These results are also reported in other studies which consider *Giardia* the most common enteric parasite of dogs and cats in developed countries [2, 3, 23, 28, 44–48]. In shepherd dogs, hookworms were the most prevalent parasite species detected.

Among the targets of this study was to investigate the potential effect of animal lifestyle to parasitism so animals living in households, shelters or farms were included. The results showed that more than half of the shelter dogs and cats were infected with at least one species of endoparasite, which was more or less expected, taking into consideration the less hygienic conditions that those animal are living in combined with a high population density that usually exists in shelters. A high level of parasitism has been previously reported in shelter dogs [3, 5, 22, 24] while in shelter cats the prevalence observed in other studies was lower [49, 50].

More than half of the shepherd dogs (51.4%) were positive for at least one species of intestinal parasite. The infection rate of intestinal parasites estimated in shepherd dogs in this study was higher than in a previous record from Greece (26.0%) [27]. Such differences are expected in cross-sectional studies especially given time and region differences. However, our results were in agreement with a study conducted in farm dogs in Portugal (57.4%) [51]. Shepherd/farm dogs often receive less veterinary care and preventive treatments. Compared to a general average of more than 2 anthelmintic treatments per year, shepherd dogs in our study received only 0.5 treatments per year.

Although the prevalence of intestinal parasites in household dogs was lower than in shelters, although not statistically significant for many species, the percentage of individuals infected was still noteworthy (23.8%). In similar studies conducted in Italy the prevalence of intestinal parasites in household dogs was even higher, reaching 57.0% of the animals [6, 23]. In our study there was no difference in the risk of infection between dogs living in an apartment with no access to a yard or a garden and dogs living in a house with access to outdoors. A reason for that could be that even dogs that are kept permanently indoors are regularly being walked by their owners in public places getting in close contact with other dogs (including stray ones) or their contaminated faeces. Similar results were recorded in the household cats studied, but this was probably due to the fact that the majority of them also live partially outdoors. The parasitism reported in household cats in this study is in agreement with the infection rates reported in Austria, Belgium, the Netherlands, France, Hungary, Italy, Romania and Spain [6, 8, 21].

Although both household and shelter dogs received regular anthelmintic treatments (i.e. an average of 2.3 for household dogs and 2.2 for shelter dogs per year), this seemed not to control parasitism efficiently. This is in agreement with the general recommendation by ESCCAP for roundworms in which it is suggested that annual or twice yearly treatments do not have a significant impact on the prevalence of patent infections within a population, and therefore a treatment frequency of at least 4 times per year is recommended (Worm Control in Dogs and Cats - ESCCAP, www.esccap.org). Recent modeling indicated that the environmental *Toxocara* contamination by dogs can only be reduced significantly if compliance to the four times a year treatment advice is sufficiently high (90%) or if at least half of the dog owners consistently remove their dog’s faeces [52]. In cats, the frequency of anthelmintic treatment differed between categories, with shelter cats being more frequently treated (i.e. an average of 1.9 for household cats and 2.7 for shelter cats per year). This could be explained by a misconception of the cat owners that indoor cats do not need preventive treatments [53].

Despite the high prevalence of parasitic infections, most animals were healthy with no obvious signs of suffering probably due to the low parasitic burden, as at least suggested by the low number of egg/(oo)cyst output recorded in most cases (even if usually there is not a clear correlation between numbers of eggs/(oo-)cysts and clinical signs). It was not statistically proven that recent records of diarrhoea were correlated to parasitism as also shown previously [54, 55] although there was evidence that anthelmintic treatment had a positive effect on reducing such records. A supporting argument for the absence of clinical disease could be that the majority of animals were adults at the time of sampling. Young animals are more sensitive to parasitism [56] but although in this study there was a tendency of older animals (> 2 year-old dogs and > 1 year-old cats) to be less infected, this was not statistically significant for most parasites. The only statistically proven facts were that the chance to get infected by *Giardia* spp. and the infection intensity of *T. leonina* was negatively correlated to age in dogs.

Given the high prevalence and the potential zoonotic importance, *Giardia* and *Cryptosporidium* positive samples were further investigated by PCR and sequencing of the positive PCR products. In dogs, the host-specific assemblages C and D dominated, which has been described before in various studies [23, 24, 36, 57–62]. Few dogs were (co)- infected with assemblage A, and the majority of these were identified as sub-assemblage AI. Sub-assemblage AI is frequently found in animals, while humans are most frequently infected with sub-assemblage AII [63, 64]. The sequence analysis in one *G. duodenalis* sample further revealed a multilocus genotype (MLG) which was previously described in calves in China [43]. Together, these results suggest that there is no significant risk for zoonotic transmission of *Giardia* infections from dogs in Crete.

In cats, the genotyping results seemed to indicate the dominance of the potentially zoonotic assemblage A in shelter animals and the co-infection of assemblages A and the feline specific assemblage F in household cats. However, the zoonotic assemblage A was identified only at the 18S rDNA locus, while only assemblage F was identified at the other loci. Since no distinction could be made between assemblages A and F in the amplified region of the conserved rRNA 18S gene, it cannot be excluded that (some of) the samples that were amplified with rDNA 18S gene were assemblage F instead of A. Therefore, no conclusion can be drawn on the zoonotic risk associated with *Giardia* infections in cats.

Regarding *Cryptosporidium*, the dog specific *C. canis* was identified in only two household dogs and the pig specific *C. scrofarum* in one shelter dog. *Cryptosporidium canis* has been also detected in household dogs in other studies [48, 65, 66] and isolated in humans, mainly children and immunocomprimised individuals in developing countries [67, 68], suggesting its potential public health impact. To our knowledge, this is the first case of *C. scrofarum* infection reported in a dog. Since keeping backyard pigs is quite a common practice in the area, it is possible that this dog ingested the oocysts before being transferred to the shelter. In such a scenario this could be a case of pseudoparasitism, given that this dog was 2.5 month-old and only recently introduced to the shelter. In cats, sequencing was not efficient; nevertheless, it revealed the presence of the feline-specific *C. felis*. Since our genotyping results revealed the presence of host-specific *Cryptosporidium* species in both dogs and cats which have been implicated in very few human infections and mainly in developing countries, we could suggest that the zoonotic potential of *Cryptosporidium* from dogs in the study area is low.

Apart from *Giardia* and *Cryptosporidium*, ascarids, hookworms and taeniids are also considered to be zoonotic [13, 69–72]. The two major ascarid species *T. canis* and *T. cati* (to a lesser extent) are responsible for human infections [13, 72]. In our study the prevalence of *Toxocara* spp. in dogs and cats was 7.6 and 8.3%, respectively. In dogs, we characterised all *Toxocara* eggs found as *Toxocara* spp. since those infections were only microscopically diagnosed and as previously suggested they could either belong to *T. canis* or *T. cati* since coprophagy is not unusual for dogs and the presence of *T. cati* eggs in dog faeces might in fact relate to pseudoparasitism [73, 74]. The infection rates found in the present study are similar to those reported in Europe which vary from 3.5 to 34.0% for *T. canis* in dogs from different epidemiological environments and from 7.2 to 76.0% for *T. cati* in cats [8, 10, 18, 52, 75–79]. The *Toxocara* infection was high, especially in shelter dogs and cats, as also reported before [24, 80, 81]. Although mainly *T. canis* is considered responsible for human toxocarosis [82], the role of *T. cati* in human toxocarosis should not be underestimated [82–84]. In Greece, toxocarosis in humans has not been studied extensively since published data are restricted only to some sporadic cases [85, 86] and one study regarding the seroprevalence of *T. canis* in children [87]. Our results combined to all European studies presented above strongly suggest that more information is needed.

Hookworm infection rates were 9.2% in dogs and 7.6% in cats. The highest infection rates of hookworms were identified in shepherd dogs (33.3%) similar to the study of Mateus et al. [51] in Portugal (31.0%). Since different hookworm species were not differentiated, the zoonotic risk associated with hookworm infections could not be determined.

The detection of taeniid eggs in shepherd dogs is worth mentioning. Unlike shelter and household dogs, shepherd dogs seem to be more prone to taeniid infection, which possibly is due to the frequent consumption of raw meat and carcasses [2, 88]. Echinococcosis is still endemic in Greece with a high prevalence reported in livestock [89–91]. However, there are no recent reports regarding the prevalence of taeniids in dogs. Taking into consideration our results in combination with the high prevalence of *E. granulosus* in livestock, which is transmitted through dogs, we could assume that shepherd dogs in Greece could be a reservoir for human infections.

## Conclusions

In conclusion, we have recorded high levels of (multi)parasitism in both dogs and cats in the study area. Most of the animals were harbouring different species of parasites sometimes in high numbers according to the egg/(oo)cyst counts. This is a proof that those parasites are greatly abundant within animal populations regardless of lifestyle. Thus, the results of our study, stress the need for better anthelmintic control schemes in dogs and cats tailored to their individual needs in order to safeguard animal and public health.

## Additional files

Additional file 1: Examples of alignments analyzed (different target genes) in both dogs and cats. (ZIP 3 kb)
Additional file 2: Table S1. Binary logistic model for *Giardia* spp. infection rate in dogs. **Table S2.** Zero-inflation negative binomial model for parasite infection intensity in dog samples. (DOCX 17 kb)
