# Supplementary material for: Abundance, zoonotic potential and risk factors of intestinal parasitism amongst dog and cat populations: The scenario of Crete, Greece
Source: Parasit Vectors. 2017 Jan 25;10:43. doi: 10.1186/s13071-017-1989-8 (PMC5264337; doi:10.1186/s13071-017-1989-8)
Supplement: Additional file 2: Table S1. — Binary logistic model for Giardia spp. infection rate in dogs. Table S2. Zero-inflation negative binomial model for parasite infection intensity in dog samples. (DOCX 17 kb) [file 13071_2017_1989_MOESM2_ESM.docx]

Additional file 1. Table S1. Binary logistic model for *Giardia* spp. infection rate in dogs

| **Variable** | **B** | **SE** | **Wald** | ***df*** | ***p*** | **OR** | **95% CI** | |
| --- | --- | --- | --- | --- | --- | --- | --- | --- |
|  |  |  |  |  |  |  | **Lower** | **Upper** |
| age | -0.019 | 0.004 | 18.225 | 1 | < 0.001 | 0.981 | 0.972 | 0.990 |
| type: *shelter^a^* |  |  | 69.533 | 2 | < 0.001 |  |  |  |
| type: *household* | -2.418 | 0.336 | 51.823 | 1 | < 0.001 | 0.089 | 0.046 | 0.172 |
| type: *shepherd* | -2.743 | 0.611 | 20.149 | 1 | < 0.001 | 0.064 | 0.019 | 0.213 |
| othanim: *no other animal^a^* |  |  | 10.804 | 3 | 0.013 |  |  |  |
| othanim: *dog* | 0.395 | 0.311 | 1.618 | 1 | 0.203 | 1.485 | 0.807 | 2.730 |
| othanim: *cat* | -1.002 | 1.051 | 0.909 | 1 | 0.340 | 0.367 | 0.047 | 2.881 |
| othanim: *mix* | -0.564 | 0.400 | 1.983 | 1 | 0.159 | 0.569 | 0.260 | 1.247 |
| Constant | 1.046 | 0.402 | 6.764 | 1 | 0.009 | 2.845 |  |  |
| **Notes**^a^ Set to zero because it was redundant  R^2^ (Nagelkerke): 34.4%  Omnibus Tests of Model Coefficients: *χ*^2^ (6) = 201.586, *p*< 0.001  H-L test: *χ*^2^ (8) = 4.309, *p* = 0.828 | | | | | | | | |

**Additional file 1. Table S2.** Zero-inflation negative binomial model for parasite infection intensity in dog samples

1. *Giardia* spp. cysts per gram

| *Count Component* | | | | | |
| --- | --- | --- | --- | --- | --- |
| **Variable** | **B** | **SE** | ***z*** | ***p*** | **OR** |
| Intercept | 9.991 | 0.135 | 73.983 | < 0.001 | 21829.117 |
| type:*Household* | 0.471 | 0.248 | 1.901 | 0.057 | 1.602 |
| log(theta) | -0.995 | 0.095 | -10.475 | < 0.001 | 0.370 |
| *Zero-inflation Component* | | | | | |
| Intercept | -0.648 | 0.158 | -4.103 | < 0.001 | 0.523 |
| age | 0.019 | 0.004 | 4.623 | < 0.001 | 1.019 |
| type:*Household* | 1.980 | 0.188 | 10.531 | < 0.001 | 7.243 |
| **Notes**  Log-likelihood: -2599 on 6 *df*  The logit link function was used in the zero-inflation component | | | | | |

1. *Cryptosporidium* spp. oocysts per gram

| *Count Component* | | | | | |
| --- | --- | --- | --- | --- | --- |
| **Variable** | **B** | **SE** | ***z*** | ***p*** | **OR** |
| Intercept | 5.792 | 0.184 | 31.445 | < 0.001 | 327.668 |
| type:*Household* | 2.500 | 0.400 | 6.247 | < 0.001 | 12.182 |
| log(theta) | -0.329 | 0.191 | -1.724 | 0.085 | 0.720 |
| *Zero-inflation Component* | | | | | |
| Intercept | 1.74 | 0.170 | 10.246 | < 0.001 | 5.697 |
| type:*Household* | 2.11 | 0.349 | 6.049 | < 0.001 | 8.248 |
| **Notes**  Log-likelihood: -549.2 on 5 *df*  The logit link function was used in the zero-inflation component | | | | | |

1. *T. leonina* eggs per gram

| *Count Component* | | | | | |
| --- | --- | --- | --- | --- | --- |
| **Variable** | **B** | **SE** | ***z*** | ***p*** | **OR** |
| Intercept | 7.026 | 0.460 | 15.262 | < 0.001 | 1125.520 |
| age | -0.073 | 0.016 | -4.682 | < 0.001 | 0.930 |
| log(theta) | -0.949 | 0.413 | -2.297 | 0.022 | 0.387 |
| *Zero-inflation Component* | | | | | |
| Intercept | 0.998 | 0.095 | 10.532 | < 0.001 | 2.713 |
| type:*Household* | 0.465 | 0.137 | 3.398 | 0.001 | 1.592 |
| **Notes**  Log-likelihood: -549.2 on 5 *df*  The clog-log link function was used in the zero-inflation component | | | | | |
